# Domestic

**Published:** 1841-02-27

**Authors:** 


### DOMESTIC.

*Weekly Report of Interments in the City and County of New York, from the 13th to the 20th of Feb.*, 1841.—Diseases: Apoplexy, 3; asphyxia, 2; bleeding, 1; burned or scalded, 1; casualties, 1; cancer, 1; colic, 2; consumption, 30; convulsions, 9; croup or hives, 3; delirium tremens, 3; diarrhœa, 1; dropsy, 1; dropsy in the head, 8; dropsy in the chest, 3; erysipelas, 1; epilepsy, 1; fever, scarlet, 11; do. typhoid, 6; do. puerperal, 1; do. bilious, 1; intemperance, 2; inflammation of brain, 2; do. of liver, 1; do. of chest, 4; do. of lungs, 16; do. of bowels, 9; do. of bladder, 1; jaundice, 1; killed or murdered, 1; marasmus, 10; measles, 1; old age, 5; organic disease of heart, 2; palsy, 2; rheumatism, 1; sprue, 1; small pox, 4; scrofula, 2; teething, 2; unknown, 3; varioloid, 1; whooping cough, 1.

Ages—Of 1 year and under, 39; between 1 and 2, 22; 2 and 5, 20; 5 and 10, 4; 10 and 20, 9; 20 and 30, 16; 30 and 40, 22; 40 and 50, 4; 50 and 60, 9; 60 and 70, 11; 70 and 80, 4; 80 and 90, 2.

34 men—36 women—57 boys—35 girls. Total, 162.

### HEALTH OF THE CITY.

INTERMENTS *in the City and Liberties of Philadelphia, from the 13th to the 20th of February,* 1841.

| Diseases. | Adults. | Children. |
|---|---|---|
| Apoplexy, | 2 | 0 |
| Cancer, | 1 | 0 |
| —— stomach, | 1 | 0 |
| Croup, | 0 | 4 |
| Cong. of brain, | 0 | 1 |
| Consumption of the lungs, | 15 | 2 |
| Contusion, | 1 | 0 |
| Convulsions, | 0 | 5 |
| Dyspepsia, | 1 | 0 |
| Diarrhœa, | 1 | 0 |
| Dropsy, | 1 | 0 |
| —— abdominal, | 0 | 1 |
| —— head, | 0 | 3 |
| Dysentery, | 1 | 0 |
| Debility, | 0 | 2 |
| Erysipelas, | 1 | 0 |
| Effusion on brain, | 0 | 1 |
| Fever, scarlet, | 0 | 2 |
| Hernia, | 0 | 1 |
| Hæmorrhage, | 0 | 1 |
| Inflammation of the brain, | 2 | 0 |
| —— bronchi, | 0 | 6 |
| —— lungs, | 3 | 5 |
| —— bowels, | 1 | 3 |
| —— kidneys, | 1 | 1 |
| —— breast, | 0 | 1 |
| Marasmus, | 1 | 1 |
| Measles, | 0 | 1 |
| *Carried forward,* | 33 | 41 |

| Diseases. | Adults. | Children. |
|---|---|---|
| *Brought forward,* | 33 | 41 |
| Mania a Potu, | 1 | 0 |
| Old age, | 2 | 0 |
| Palsy, | 1 | 0 |
| Pneumothorax, | 1 | 0 |
| Scrofula, | 0 | 1 |
| Small pox, | 2 | 1 |
| Still-born, | 0 | 10 |
| Ulcers, | 1 | 0 |
| Ulcerated sore throat, | 0 | 2 |
| Unknown, | 2 | 0 |
| Total, 98— | 43 | 55 |

Of the above, there were under 1 year, 33

| | |
|---|---|
| From 1 to 2 | 8 |
| 2 to 5 | 9 |
| 5 to 10 | 2 |
| 10 to 15 | 1 |
| 15 to 20 | 2 |
| 20 to 30 | 7 |
| 30 to 40 | 9 |
| 40 to 50 | 9 |
| 50 to 60 | 6 |
| 60 to 70 | 6 |
| 70 to 80 | 4 |
| 80 to 90 | 2 |
| 90 to 100 | 0 |
| Total, | 98 |

In the above are included 15 people of colour, and 4 interments from the alms-house.

